# Exercise training improves diabetic renal injury by reducing fetuin-A, oxidative stress and inflammation in type 2 diabetic rats

**DOI:** 10.1016/j.heliyon.2024.e27749

**Published:** 2024-03-12

**Authors:** Shadan Saberi, Majid Askaripour, Mohammad Khaksari, Mohammad Amin Rajizadeh, Mohammad Abbas Bejeshk, Mohammad Akhbari, Elham Jafari, Kayvan Khoramipour

**Affiliations:** a Physiology Research Center, Institute of Neuropharmacology, Kerman University of Medical Sciences, Kerman, Iran; b Department of Physiology, School of Medicine, Bam University of Medical Sciences, Bam, Iran; c Endocrinology and Metabolism Research Center, Kerman University of Medical Sciences, Kerman, Iran; d Cardiovascular Research Center, Institute of Basic and Clinical Physiology Sciences, Kerman University of Medical Sciences, Kerman, Iran; e Student Research Committee, Kerman University of Medical Sciences, Kerman, Iran; f Department of Pathology, Pathology and Stem Cell Research Center, Afzalipour Faculty of Medicine, Kerman University of Medical Sciences, Kerman, Iran; g Neuroscience Research Center, Institute of Neuropharmacology, Kerman University of Medical Sciences, Kerman, Iran

**Keywords:** Diabetic kidney disease, High-intensity interval training, Inflammation, Oxidative stress, Fetuin-A, Kim-1

## Abstract

**Background:**

Diabetic kidney disease (DKD) stands as a primary contributor to end-stage renal disease, associated with heightened mortality in cardiovascular diseases. This study aimed to explore the impact of an eight-week high-intensity interval training (HIIT) on renal injury in diabetic rats.

**Methods:**

Twenty-eight male Wistar rats were randomly allocated into four groups: healthy control (CTL), diabetic control (DC), exercise (EX), and diabetes-exercise (D + EX). Induction of diabetes in the DC and D + EX groups occurred through a two-month high-fat diet followed by a single dose of 35 mg/kg streptozotocin (STZ). Rats in the EX and D + EX groups underwent 4–10 intervals of HIIT (80–100% Vmax) over 8 weeks. Subsequently, pathological and biochemical parameters were assessed in the serum and kidney tissue of the experimental groups.

**Results:**

In the DC group, diabetes led to elevated kidney damage, glomerulosclerosis, fasting blood glucose (FBG), Homeostatic Model Assessment for Insulin Resistance (HOMA-IR) index, animal weight, kidney dysfunction, albuminuria, and glomerular filtration rate. Additionally, serum and kidney levels of fetuin-A increased, along with kidney levels of KIM-1. Mechanistically, diabetes induction resulted in kidney inflammation by elevating levels of tumor necrosis factor-alpha (TNF-α), transforming growth factor beta (TGF-β), and interleukin 6 (IL-6), while reducing IL-10 levels and increasing the IL-6/IL-10 ratio. Furthermore, diabetes triggered renal oxidative stress, evidenced by increased Malondialdehyde (MDA) levels and decreased levels of glutathione peroxidase (GPx), catalase, and superoxide dismutase (SOD). HIIT mitigated the adverse effects of diabetes in the D + EX group compared to the DC group.

**Conclusion:**

Our findings suggest that HIIT ameliorates type 2 diabetes (T2D)-induced kidney damage by mitigating inflammation, lowering serum levels of fetuin-A, and bolstering antioxidant defenses. This study highlights the potential of HIIT as a time-efficient intervention for diabetic nephropathy**.**

## Graphical abstract

**Figure:**
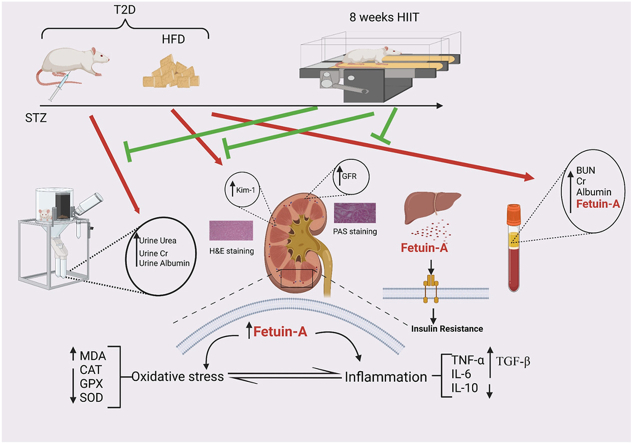

## 1 Introduction

An inactive lifestyle is widely recognized as the primary contributor to metabolic disorders, including diabetes [1,2]. According to the latest report from the International Diabetes Federation, approximately 463 million people worldwide are living with diabetes, a number projected to escalate to 700 million by 2045 [3,4]. Diabetic kidney disease (DKD) affects around 30–40% of diabetic patients, presenting as one of the most prevalent microvascular complications of diabetes and contributing to increased mortality from cardiovascular diseases [5,6]. Additionally, it stands as a leading cause of end-stage renal disease (ESRD) [7].

Experimental investigations have outlined the initial phases of diabetic kidney disease, characterized by high blood sugar levels, increased glomerular filtration rate, renal adaptive hypertrophy, and microalbuminuria. These changes arise from the hemodynamic and metabolic disruptions linked to diabetes [8]. DKD induces diverse morphological changes in different kidney components, encompassing glomerulosclerosis, basement membrane damage, extracellular matrix accumulation, and tubular injury, along with escalated levels of tubular injury biomarkers such as kidney injury molecule-1 (KIM-1) [[8], [9], [10]]. KIM-1, a type 1 transmembrane protein, typically exhibits minimal expression in healthy kidneys but undergoes upregulation in various human kidney diseases or animal models during acute and chronic phases [11,12].

Fetuin-A, a glycoprotein cytokine predominantly released from the liver (over 95%) and adipose tissue in adults, plays a crucial role in insulin resistance and the pathogenesis of type 2 diabetes (T2D) [13,14]. Notably, non-diabetic dialysis patients have shown a robust association between fetuin-A levels and abdominal obesity and dyslipidemia [15]. Furthermore, fetuin-A levels exhibit variations among individuals with chronic kidney disease, with lower levels observed in those with ESRD compared to healthy individuals or those with mild to moderate kidney disease [16]. Interestingly, high fetuin-A levels in healthy diabetic and non-diabetic individuals without vascular disease history correlate with increased vascular risks, yet an inverse relationship is observed in individuals with existing vascular issues [17]. Additionally, fetuin-A, functioning as a positive acute-phase protein, stimulates the expression of inflammatory cytokines in adipocytes and macrophages, thereby serving as an inflammatory marker [18].

Exercise training emerges as a safe, non-pharmacological approach in the prevention and management of various metabolic disorders and their complications. Numerous studies have illustrated the diverse effects of exercise in reducing mortality risk and halting kidney disease progression [[19], [20], [21]]. The efficacy of exercise hinges on various factors, including intensity and duration [[22], [23], [24]]. Notably, high-intensity interval training (HIIT) has demonstrated superior enhancements in insulin sensitivity, mitochondrial density, oxidative enzymes, lung function, immune function, and cardiac output compared to other exercise modalities [22,23].

This study aims to scrutinize the effects of HIIT on renal histopathology and function in type 2 diabetic rats, evaluating serum and tissue levels of fetuin-A alongside renal markers of inflammation and antioxidant defense.

## 2 Material and methods

### 2.1 Animals

Twenty-eight male Wistar rats with an average weight of 200 ± 20 g was purchased from the animal farm of Kerman University of Medical Sciences and kept in special cages at a temperature of 23 ± 1°^C^, a humidity of 46–54% and a light-dark cycle of 12:12 h. During the study, the animals had free access to water and food. Of the whole study procedure was approved by Ethics Committee of Kerman University of Medical Sciences (Ethic code: IR.KMU.REC.1400.439.).

### 2.2 Experimental groups and protocols

**Fig. 1:**
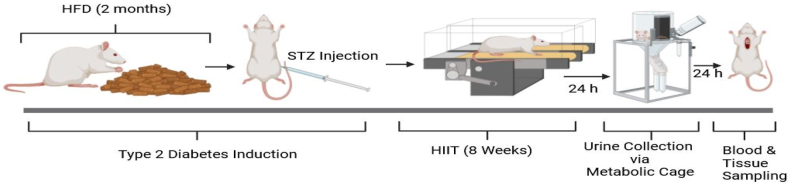
Time-line for study procedures.

After acclimatization to the laboratory environment, the animals were randomly assigned to one of four groups: healthy control (CTL), diabetic control (DC), exercise (EX), and diabetes-exercise (D + EX), with each group consisting of seven rats (n = 7). We lost some rats (5 rats) but replaced them to have the desired sample size. T2D was induced in the DC and D + EX groups by administering a two-month high-fat diet along with a single intraperitoneal injection of 35 mg/kg streptozotocin (STZ) (Sigma Aldrich). Fasting blood glucose (FBG) levels were measured three days post-STZ injection (0.5–1 ml blood sample was taken from the tail vein), and rats exhibiting FBG levels exceeding 300 mg/dl were classified as diabetic. Following diabetic induction, all groups were maintained on a normal diet throughout the study period. Rats in the training groups underwent an eight-week regimen of HIIT. The composition of the high-fat diet and the HIIT protocol have been detailed in our previous publications [25]. In brief, HIIT sessions comprised 4–10 intervals of running on a treadmill with no incline, alternating between 2 min of high intensity and 1 min of low intensity, with intensity set at 80–100% of the individual maximum speed (Vmax) for rats [26]. A diet comprising 60% fat, 20% protein, and 20% carbohydrate was utilized as the high-fat diet (Royan Co, Isfahan, Iran) [27]. Changes in weight and FBG levels were monitored throughout the experimental period in all groups. After the final training session, animals were individually placed in metabolic cages for 24 h to collect urine for determination of urine volume and glomerular filtration rate (GFR). GFR was calculated using the formula: [urine creatinine × urine volume]/serum creatinine [28]. Subsequently, animals were anesthetized with an intraperitoneal injection of a Ketamine/Xylazine cocktail (80/10 mg) and blood was collected from the heart, followed by centrifugation at 4000 revolutions per minute (RPM) for 15 min to obtain serum for biochemical assays. Animals were then euthanized, and tissues were harvested. The right kidneys were preserved in formalin for pathological evaluation, while the left kidneys were stored at −80 °C for biochemical analyses (Fig. 1).

### 2.3 Histopathological analysis

The right kidneys were immersed in 10% formalin for fixation, and the extent of kidney damage, encompassing tubular hydropic degradation, desquamation, brush border loss, and peritubular infiltration, was assessed using hematoxylin and eosin (H&E) staining. Histopathological evaluation of kidney injury was graded on a scale from 0 to 3: 0 denoting normal histology, 1 indicating mild injury, 2 representing moderate injury, and 3 signifying severe injury. Additionally, glomerular sclerosis was evaluated through Periodic acid Schiff (PAS) staining and scored from 0 to 3: 0 indicating normal glomerular architecture, 1 representing involvement of less than 25% of the glomerular area, 2 indicating involvement of 25–50% of the glomerular area, and 3 indicating involvement of more than 75% of the glomerular area [29].

### 2.4 Insulin resistance index and biochemical analysis

Fasting serum insulin and glucose concentrations were utilized to calculate the homeostasis model assessment index for insulin resistance (HOMA-IR) employing the following formula [30]: HOMA-IR index = [fasting glucose (mg/dl) × fasting insulin (mU/ml)]/405.

Levels of albumin, creatinine, and urea were quantified in urine samples using commercial kits. Similarly, levels of fetuin-A, albumin, creatinine, and blood urea nitrogen (BUN) were determined in serum utilizing commercial kits.

Furthermore, levels of KIM-1 (Bio-Techne Kit, RKM100), fetuin-A (Novusbio Kit, NBP2-78752), oxidant (malondialdehyde, MDA) (NavandSalamat Co), antioxidant (glutathione peroxidase (GPx) (NavandSalamat Co), catalase (NavandSalamat Co), and superoxide dismutase (SOD) (NavandSalamat Co), as well as inflammatory markers (tumor necrosis factor-alpha (TNF-α) (DuoSet Kit, DY510-05), transforming growth factor-beta (TGF-β) (Bio-Techne Kit, MB100B), interleukin-6 (IL-6) (DuoSet Kit, DY506-05), and the anti-inflammatory marker interleukin-10 (IL-10) (DuoSet Kit, DY522-05), were quantified in kidney tissue using enzyme-linked immunosorbent assay (ELISA) kits [15,31].

### 2.5 Statistical analysis

The statistical analyses were performed using GraphPad Prism 9.0 software (GraphPad Inc., La Jolla, CA, United States). Data are expressed as means ± SEM. The normality and homogeneity of variances were checked using the Shapiro–Wilk and Leven's tests, respectively. Two-way ANOVA was used to compare the differences in mean values among groups followed by Tukey's post hoc test. Correlations were evaluated using Pearson's correlation coefficient. P < 0.05 was deemed statistically significant.

## 3 Results

### 3.1 Changes in FBG and HOMA-IR index

**Fig. 2:**
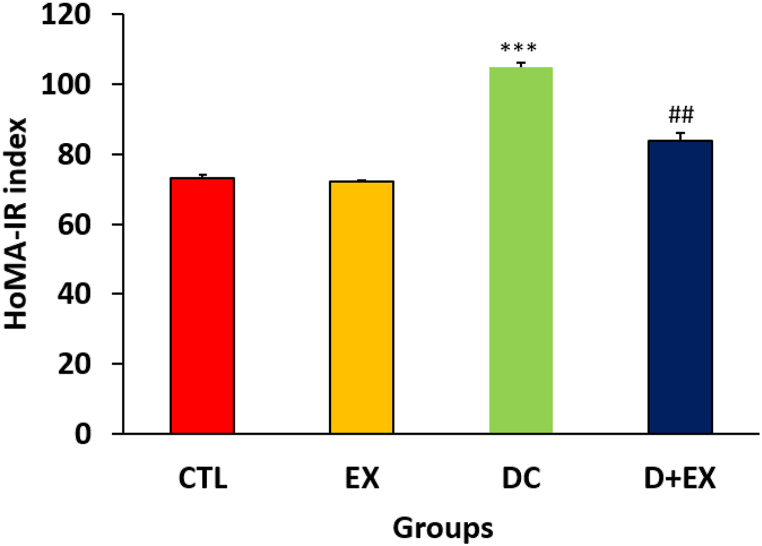
HOMA-IR (homeostasis model assessment of Insulin resistance) in experimental groups. Data are shown as Mean ± SEM. ***p < 0.001 as compared to CTL group. ##p < 0.01 compared to DC group. CTL, control; EX, exercise; DC, diabetic control; D + EX, diabetes-exercise.

According to Table 1, after induction of diabetes (month 2) the levels of FBG in the DC and D + EX groups increased significantly compared to the baseline (p < 0.001). HIIT reduced the levels of FBG significantly (p < 0.001, month 4). As expected, HOMA-IR (Fig. 2) increased in diabetic rats in comparison with the control group (p < 0.05). HIIT significantly reduced HOMA-IR in the D + EX compared to the DC group (P < 0.01).

**Table 1:** Fasting blood glucose of experimental groups.

Groups	Time (month)		
	0	2	4
**CTL**	221.8 ± 1.16	224.6 ± 0.93	222.3 ± 1.18
**EX**	224.7 ± 1.70	224 ± 1.30	225 ± 1.58
**DC**	225.2 ± 0.84	379.2 ± 1.69***	384.6 ± 1.72***
**D** + **EX**	225.2 ± 0.86	385 ± 1.97***	265.9 ± 1.88 ###

Data are shown as Mean ± SEM. ***p < 0.001 significant difference in month 2 compared with baseline (month 0). ###p < 0.001 significant difference between month 4 and month 2 in D + EX group. CTL, control; EX, exercise; DC, diabetic control; D + EX, diabetes-exercise (n = 7).

### 3.2 Changes in animal weight during experimental period

Based on Table 2, receiving two months HFD and a single dose of STZ increased the animal's weight in DC groups significantly in compared to baseline (month 0). 8 weeks HIIT prevented animals' weight change in EX group compared to beginning of exercise (month 2), whilst untreated DC group revealed weight loss in remainder period of the study.

**Table 2:** Animal weight of experimental groups.

Groups	Time (month)		
	0	2	4
**CTL**	185.3 ± 2.7	204.2 ± 1.4	219.8 ± 2.5
**EX**	196.3 ± 2.7	216.2 ± 3.2	219.6 ± 2.5
**DC**	183.3 ± 2.3	259.6 ± 2.5***	166.8 ± 2.6 ###
**D** + **EX**	195.3 ± 1.8	256.9 ± 2.7***	260 ± 2.1***

Data are shown as Mean ± SEM. ***p < 0.001 shows a significant difference with baseline (month 0). ###p < 0.001 significant difference between month 4 and month 2 in DC group. CTL, control; EX, exercise; DC, diabetic control; D + EX, diabetes-exercise.

### 3.3 HIIT improved renal function, urine volume and GFR of diabetic rats

**Fig. 3:**
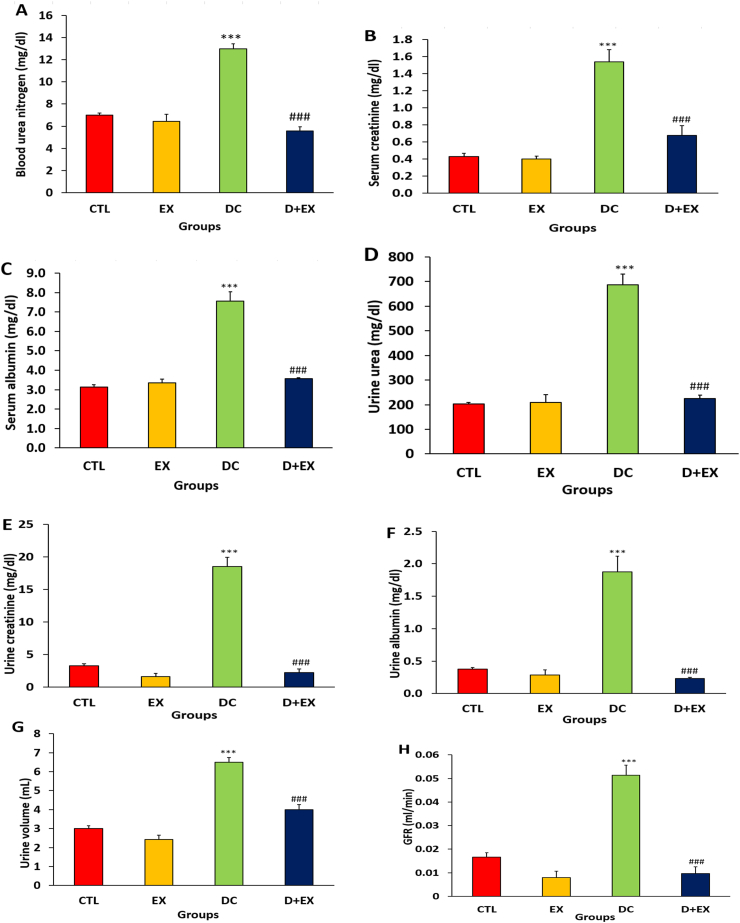
Blood urea nitrogen (**A**), serum creatinine (**B**), serum albumin (**C**), urine urea (**D**), urine creatinine (**E**), urine albumin (**F**), urine volume (**G**) and GFR (**H**) in experimental groups. Data are shown as Mean ± SEM. ***p < 0.001 as compared to CTL group. ###p < 0.001 compared to DC group. CTL, control; EX, exercise; DC, diabetic control; D + EX, diabetes-exercise.

Induction of T2D increased the levels of urea, creatinine and albumin in the serum and urine of the DC group compared to the control group significantly (p < 0.001, Fig. 3A–F). These factors reduced in DC + EX compared to the DC group (p < 0.001, Fig. 3A–F)**.** The levels of urine volume and GFR increased significantly in DC group (p < 0.001, Fig. 3 G, H)**.** HIIT in diabetic group reversed these alterations (p < 0.001, Fig. 3 G, H).

### 3.4 HIIT improved renal histopathological damage of diabetic rats

**Fig. 4:**
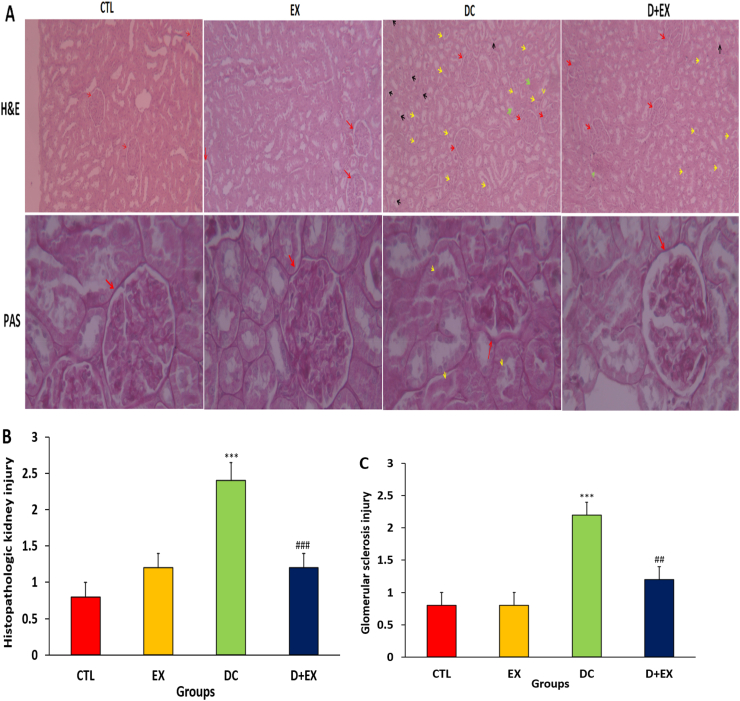
Representative sections stained by H&E ( × 100 magnification, Scale bar = 200 μm) and PAS ( × 400 magnification, Scale bar = 20 μm) methods of kidney in experimental groups (**A**). Semi-quantitative score of kidney injury (**B**) and glomerular sclerosis injury (**C**). Data are shown as Mean ± SEM. ***p < 0.001 as compared to CTL group. ##p < 0.01, ###p < 0.001 compared to DC group. CTL, control; EX, exercise; DC, diabetic control; D + EX, diabetes-exercise. In the H&E staining red arrows is normal architecture and glomeruli in the CTL and EX groups. In DC group show sever kidney injury includes glomerular damage (red arrows), inflammatory cells infiltration, congestion (green arrow), marked desquamation (black arrows), loss of brush border, and swelling of tubule cells and some tubular casts (yellow arrow).in the D + EX kidney injury is less than DC group includes glomerular damage (red arrows), inflammatory cells infiltration, congestion (green arrow), marked desquamation (black arrows), loss of brush border, swelling of tubule cells and some tubular casts (yellow arrow). In the PAS staining, red arrow is normal glomeruli in CTL, EX and D + EX groups. A glomerulus with areas of segmental sclerosis (red arrow) and some tubular casts (yellow arrow) were shown in DC group. (For interpretation of the references to colour in this figure legend, the reader is referred to the Web version of this article.)

Histopathologic kidney injury and glomerular sclerosis injury were significantly higher in the DC group compared to the CTL group (P < 0.001, Fig. 4A–C). In the D + EX groups, HIIT, decreased the kidney injury score and glomerular sclerosis compared to the DC group (p < 0.001).

### 3.5 The effects of HIIT on serum and kidney levels of fetuin-A and kidney level of KIM-1

**Fig. 5:**
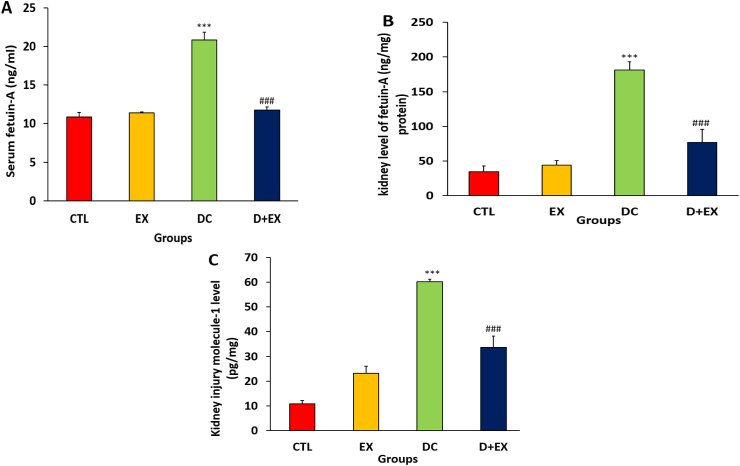
Serum level of fetuin-A (**A**), kidney level of fetuin-A (**B**) and kidney injury molecule 1 (KIM-1) level **(C)** in experimental groups. Data are shown as Mean ± SEM. ***p < 0.001 as compared to CTL group. ###p < 0.001 compared to T2D group. CTL, control; EX, exercise; DC, diabetic control D + EX, diabetes-exercise. Data are shown as Mean ± SEM. ***p < 0.001 as compared to CTL group. ###p < 0.001 compared to DC group. CTL, control; EX, exercise; DC, diabetic control; D + EX, diabetes-exercise.

The serum and kidney levels of fetuin-A are shown in Fig. 5 A, B. The fetuin-A in the serum and kidney tissue of diabetic rats significantly increased compared to the CTL group (p < 0.001). HIIT in diabetic rats decreased the serum and kidney levels of fetuin-A in compared to DC group (p < 0.001). Based on our results, the kidney level of KIM-1 in the DC group significantly increased compared to the control group (p < 0.001, Fig. 5 C)**.** In the D + EX groups, HIIT, decreased the level of this marker compared to the DC group (p < 0.001).

### 3.6 The effects of HIIT on renal oxidative stress of diabetic rats

**Fig. 6:**
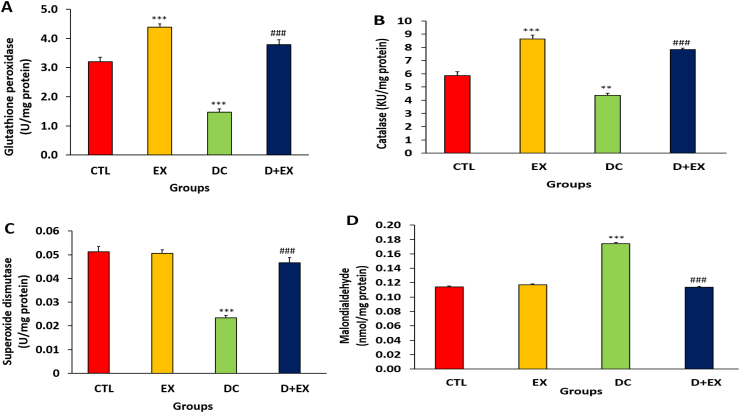
Effects of HITT on oxidative stress biomarkers in the kidney of diabetic rats. Glutathione peroxidase (**A**), catalase (**B**), superoxide dismutase (**C**) and malondialdehyde (**D**). Data are shown as Mean ± SEM. **p < 0.01, ***p < 0.001 as compared to CTL group. ###p < 0.001 compared to DC group. HITT, high-intensity interval training; CTL, control; EX, exercise; DC, diabetic control; D + EX, diabetes-exercise.

In compared to control group, the kidney levels of antioxidant enzymes activity including glutathione peroxidase, catalase and superoxide dismutase decreased significantly and the kidney level of malondialdehyde increased significantly in DC group (p < 0.05, Fig. 6A–D). In the diabetic rats, HIIT reversed the levels of these markers compared to the DC group (p < 0.001)**.**

### 3.7 The effects of HIIT on renal inflammation markers of diabetic rats

**Fig. 7:**
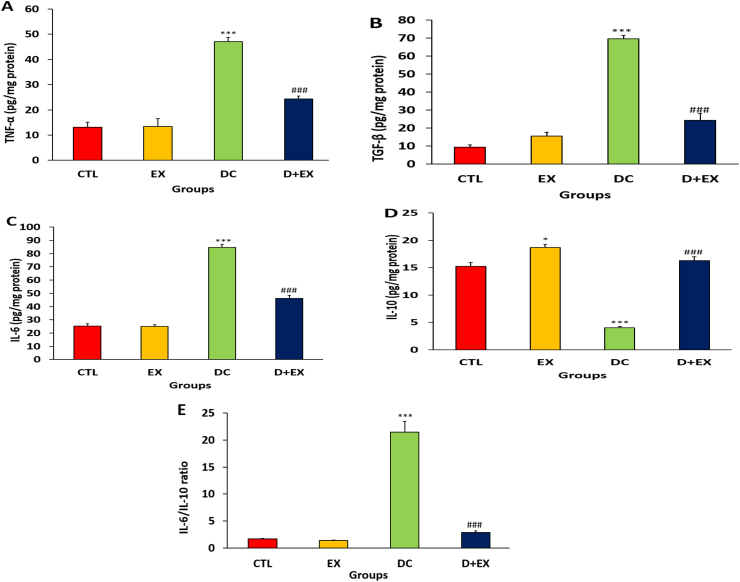
Effects of HITT on inflammation markers in the kidney of diabetic rats. TNF-α (A), TGF-β (B), IL-6 (C), IL-10 (D) and IL-6/IL-10 ratio (E).Data are shown as Mean ± SEM. *p < 0.05, ***p < 0.001 as compared to CTL group. ###p < 0.001 compared to DC group. HITT, high-intensity interval training; TNF-α, tumor necrosis factor alpha; IL-6, interleukin 6; IL-10, interleukin 10; CTL, control; EX, exercise; DC, diabetic control; D + EX, diabetes-exercise.

Based on the results presented in Fig. 7 A-E, the kidney levels of pro-inflammatory markers, TNF-α, TGF-β and IL-6 and ratio of IL-6/IL-10 increased and the level of anti-inflammatory IL-10 decreased significantly in DC compared to CTL group (p < 0.001). In diabetic rats, HIIT improved the inflammatory status by reversing the level of these markers compared to the DC group (p < 0.001)**.**

## 4 Discussion

This study aimed to investigate the effects of HIIT on renal histopathology and function, serum and tissue levels of fetuin-A, as well as renal markers of inflammation and antioxidant defense in T2D rats. Our data revealed that eight-week HIIT could attenuate kidney damage in an animal model of T2D (T2D induced by high-fat diet and a single dose of STZ) by improving GFR, disrupted renal structures, reducing oxidative stress, decreasing pro-inflammatory and increasing anti-inflammatory markers, and reducing KIM-1 and Fetuin-A. Furthermore, HIIT reduced FBS and HOMA-IR in T2D rats. These findings suggested that HIIT may have a therapeutic advantage for diabetic renal injury.

DKD pathogenesis is closely related to chronic inflammation and oxidative stress. Increased inflammatory cytokines levels released by adipose tissue, including TNF-α and IL-6, promote insulin resistance and correlate with the progression of diabetic nephropathy (DN) [27]. Belotto et al. [32] reported that 3 weeks of moderate exercise can reduce the levels of serum TNF-α and IL-6 in rats with T2D. In the Lin et al. study [33], low, moderate, and high-intensity training were demonstrated to reduce TNF-α and IL-6 cytokine levels in the serum of diabetic rats. Ishikawa et al. [34] also reported that in type 2 diabetic KK-A y mice, low-intensity exercise mitigates the progression of kidney damage by decreasing inflammation and oxidative stress. In the present study, HIIT reduced the level of TNF-α and IL-6 cytokine and increased IL-10 level in the kidney of diabetic rats.

Several mechanisms, including hemodynamic malfunction, hyperglycemia, glycosylation of non-enzymatic protein, and aberrant metabolism of cellular glucose, have a crucial role in the pathogenesis of DN, which lead to activation and release of IL-6 and TNF-α and reduction of IL-10 in the kidney tissue, which finally causes the accumulation and deposition of extracellular matrix in the glomerular mesangium [35]. TGF-β is a key profibrotic mediator, which has a crucial role in the pathogenesis of DN and renal inflammation [[36], [37], [38]]. It is proved that hyperglycemia in diabetes stimulates TGF-β activation [39]. In the present study, TGF-β and inflammatory cytokines were elevated in the DC group accompanied by impaired renal function (increase of serum levels of BUN, Cr, and albuminuria). HIIT reversed these values, confirming its protective role in diabetic nephropathy. In agreement, other researchers reported the renoprotective effect of 8 weeks exercise through TGF-β1 reduction in diabetic rats [40].

Progressive histological manifestation of DN includes increasing glomerular hypertrophy, thickness of the glomerular basement membrane, and increasing urinary excretion of proteins which finally results in renal failure and glomerulosclerosis [35]. Our observations confirmed these results, as histopathological kidney injury and glomerulosclerosis score were increased in addition to increased albuminuria in the DC group, while HIIT could improve renal structural changes.

Several studies demonstrated a direct relationship between oxidative stress and diabetes complications which is caused by hyperlipidemia and hyperglycemia, insulin resistance, and pancreatic β-cell dysfunction [41]. Moreover, the ROS generation is increased by inflammatory cytokines such as nuclear factor-kB (NF-kB), TNF-α, and IL-1β [42]. Our results revealed MDA elevation and antioxidant enzyme reduction in the DC group. In line with our results, Fattahi Bafghi et al. reported that HIIT increased the levels of antioxidant enzymes in heart tissue of diabetic mice [43]. In addition, aerobic exercise training improved renal function among T2D patients by decreased MDA and increased GPx and GSH [39]. In this study, the reduction of antioxidant enzyme activity including GPx, SOD, and catalase in the DC group was restored in animals underwent eight-weeks HIIT.

Our results revealed that the experimental T2D model increased kidney tissue levels of Kim-1, fetuin-A, and serum level of fetuin-A. However, eight-weeks HIIT reversed these values to near normal levels. Kim-1 is an early biomarker to diagnose renal tubular injury which its specificity and sensitivity are approved by FDA [44]. In agreement with our results, Monno et al. [45] reported that 8 weeks of exercise suppressed the levels of albuminuria, KIM-1, inflammation, and oxidative stress.

Fetuin-A gene expression and release are increased in rat hepatocytes due to receiving a high-fat diet, and patients suffering from metabolic syndrome [46,47]. Fetuin-A increased oxidative stress, inflammation, and insulin resistance [48]. Fetuin-knockout mice showed resistance to high-fat diet-induced weight gain and improved glucose and insulin tolerance [49]. Fetuin-A stimulates pro-inflammatory cytokines production, and increases TNF-α, and IL-6 expression [50]. Khadir et al. [51] reported that the serum level of fetuin-A was higher in diabetic patients, while physical exercise decreased its level. Keihanian et al. [52] revealed that 8 weeks of aerobic and resistance exercise training significantly decreased serum fetuin-A levels in males with type 2 diabetes mellitus. Some meta-analysis studies disclosed the ameliorating impacts of exercise on hyperglycemic and diabetic patients through reducing Fetuin-A. In addition, it seems that the anti-inflammatory effects of exercise following diabetes are due to Fetuin-A reduction [53]. It has been shown that in T2D with nephropathy, regular moderate exercise is a more valuable program to reduce Fetuin-A plasma level and to improve associated renal damage [54]. Several possible mechanisms explain the reduction of fetuin-A levels by performing physical activity. These include a decrease in hepatic fat and hepatic glucolipotoxicity, which are mediated by ROS formation and pro-inflammatory activation [55,56], as well as Akt activation, which can lead to an increase in glucose tolerance and a decrease in insulin resistance [57].

A direct relationship between fetuin-A and insulin resistance is known [58]. Moreover, there is an inverse relationship between fetuin-A and insulin resistance with adiponectin [[59], [60], [61]]. Our previous study with the same design showed that HIIT could increase serum adiponectin following T2D [62]. It seems that another possible mechanism of exercise to reduce fetuin-A is achieved through adiponectin signaling.

In this study, we revealed that HIIT reduced fetuin-A levels in serum and kidney tissue, which was accompanied by the elevation of anti-inflammatory cytokine IL-10, reduction of pro-inflammatory cytokine IL-6 and IL-6 to IL-10 ratio, and profibrotic cytokine TGF-β, insulin resistance, and hyperglycemia in the intervened animals. TGF-β reduction by HIIT, suggested possible beneficial effect of this kind of exercise training on renal fibrosis-induced by diabetes. The limitation of study is the lack of measurement of fibrotic biomarkers, that is suggested for future studies.

In conclusion, all the results demonstrated that the induction of T2D with a high-fat diet and a single dose of STZ (35 mg/kg) caused diabetic nephropathy through impaired insulin sensitivity, glucose metabolism, increased renal malfunction, disrupted renal structures, increased levels of inflammatory cytokines, oxidative stress, and fetuin-A. Eight-week HIIT reversed these effects. Finally, we concluded that HIIT could be a beneficial strategy to treat diabetic nephropathy. However, further studies are warranted to clarify the molecular mechanism of this effect.

## Funding

None.

## Ethic approval

Of the whole study procedure was approved by Ethics Committee of Kerman University of Medical Sciences (Ethic code: IR.KMU.REC.1400.439.).

## Availability of data and materials

The datasets used and/or analyzed during the current study are available from the corresponding author on reasonable request.

## CRediT authorship contribution statement

**Shadan Saberi:** Methodology, Investigation. **Majid Askaripour:** Writing – original draft. **Mohammad Khaksari:** Writing – review & editing. **Mohammad Amin Rajizadeh:** Methodology, Investigation. **Mohammad Abbas Bejeshk:** Investigation. **Mohammad Akhbari:** Software. **Elham Jafari:** Methodology. **Kayvan Khoramipour:** Conceptualization.

## Declaration of competing interest

The authors declare the following financial interests/personal relationships which may be considered as potential competing interests: Kayvan Khoramipour reports financial support was provided by 10.13039/501100004621Kerman University of Medical Sciences. Kayvan Khoramipour reports a relationship with Kerman University of Medical Sciences that includes: board membership. Kayvan Khoramipour has patent pending to N/A. N/A If there are other authors, they declare that they have no known competing financial interests or personal relationships that could have appeared to influence the work reported in this paper.
